# Target dose conversion modeling from pencil beam (PB) to Monte Carlo (MC) for lung SBRT

**DOI:** 10.1186/s13014-016-0661-3

**Published:** 2016-06-17

**Authors:** Dandan Zheng, Xiaofeng Zhu, Qinghui Zhang, Xiaoying Liang, Weining Zhen, Chi Lin, Vivek Verma, Shuo Wang, Andrew Wahl, Yu Lei, Sumin Zhou, Chi Zhang

**Affiliations:** Department of Radiation Oncology, University of Nebraska Medical Center, Omaha, NE USA; Department of Radiation Medicine, Northwell Health, New York, NY USA; University of Florida Health Proton Therapy Institute, Jacksonville, FL USA; School of Biological Sciences, University of Nebraska Lincoln, 1901 Vine Street, Lincoln, NE 68588-0660 USA

**Keywords:** Lung SBRT, Monte Carlo, Prescription, Target dose variation

## Abstract

**Background:**

A challenge preventing routine clinical implementation of Monte Carlo (MC)-based lung SBRT is the difficulty of reinterpreting historical outcome data calculated with inaccurate dose algorithms, because the target dose was found to decrease to varying degrees when recalculated with MC. The large variability was previously found to be affected by factors such as tumour size, location, and lung density, usually through sub-group comparisons. We hereby conducted a pilot study to systematically and quantitatively analyze these patient factors and explore accurate target dose conversion models, so that large-scale historical outcome data can be correlated with more accurate MC dose without recalculation.

**Methods:**

Twenty-one patients that underwent SBRT for early-stage lung cancer were replanned with 6MV 360° dynamic conformal arcs using pencil-beam (PB) and recalculated with MC. The percent D95 difference (PB-MC) was calculated for the PTV and GTV. Using single linear regression, this difference was correlated with the following quantitative patient indices: maximum tumour diameter (MaxD); PTV and GTV volumes; minimum distance from tumour to soft tissue (dmin); and mean density and standard deviation of the PTV, GTV, PTV margin, lung, and 2 mm, 15 mm, 50 mm shells outside the PTV. Multiple linear regression and artificial neural network (ANN) were employed to model multiple factors and improve dose conversion accuracy.

**Results:**

Single linear regression with PTV D95 deficiency identified the strongest correlation on mean-density (location) indices, weaker on lung density, and the weakest on size indices, with the following R^2^ values in decreasing orders: shell2mm (0.71), PTV (0.68), PTV margin (0.65), shell15mm (0.62), shell50mm (0.49), lung (0.40), dmin (0.22), GTV (0.19), MaxD (0.17), PTV volume (0.15), and GTV volume (0.08). A multiple linear regression model yielded the significance factor of 3.0E-7 using two independent features: mean density of shell2mm (*P* = 1.6E-7) and PTV volume (*P* = 0.006). A 4-feature ANN model slightly improved the modeling accuracy.

**Conclusion:**

Quantifiable density features were proposed, replacing simple central/peripheral location designation, which showed strong correlations with PB-to-MC target dose conversion magnitude, followed by lung density and target size. Density in the immediate outer and inner areas of the PTV showed the strongest correlations. A multiple linear regression model with one such feature and PTV volume established a high significance factor, improving dose conversion accuracy.

## Background

Accuracy of dose calculations has long been recognized as a critical issue for stereotactic body radiotherapy (SBRT) of the lung, where the heterogeneous tumour-lung interface and the large fractional dose necessitate advanced dose algorithms [1–8]. With the advent of fast Monte Carlo (MC) algorithms in major treatment planning systems during the past decade, many studies have been conducted on dose comparisons between MC and other algorithms for lung SBRT [9–32]. From these studies, it has been shown that the major dose discrepancy between different algorithms exists on the treatment target rather than normal tissues. On the other hand, very large variations in target doses have been calculated for individual cases, making a simple correction on the prescription dose using historical and inaccurate algorithms difficult. For example, a study by van der Voort van Zyp et al. showed patient-to-patient variations of 3 to 33 % in the reduction of the planning target volume (PTV) D95 by the MC compared with the original equivalent path length (EPL) calculation [17]. Similar large variations have been well described by many other reports [16, 19, 22, 24, 28], even as large as ranging from 2.9 to 82.7 % in a study by Liu et al. [18]. These studies all concluded that, from one type of algorithm to another, it is impossible to simply convert treatment protocols owing to the large observed variations.

Some studies have also found that a number of patient and treatment characteristics contribute to these variations, such as tumour size, tumour location, lung density, beam energy, prescription isodose line, and delivery technique [14, 16, 17, 19, 28, 30, 31]. Van der Voort van Zyp et al. [17] investigated the effects of various factors, and recommended different prescription doses based on tumour location and size. In the study, dose differences were compared between EPL and MC on 53 patients, and the authors demonstrated using multivariate regression analysis that the minimum distance to soft tissue and the GTV size were associated with the magnitude of dose reduction. Therefore, they recommended different MC prescription doses based on tumour location and size using the median reductions of their subgroups: a lower prescription (i.e. a larger dose reduction) for a peripheral than for a central tumour; or for a small than a large tumour.

In another study, Wu et al. [28] also investigated the effect of tumour location and size by grouping a series of 33 patients into central and peripheral location groups, with further subgrouping into large and small sizes within the location groups. In addition to comparing the reduction from EPL to MC on target dose coverage indices such as PTV D95 and D98, the authors also compared four point doses at the PTV periphery. Interestingly, for central vs. peripheral tumours, statistically significant differences did not exist for two of the four point doses, nor the PTV D95 or D98. Between the small and large tumour sizes, only a few dose-volume indices showed statistically significant differences within the peripheral tumour group, and none of the indices did so within the central tumour group. Using phantom studies, a few groups have reported the association of lung densities with the algorithm-related magnitude of dose differences in the target [16, 30]. However, such an association has heretofore not been demonstrated by studies on patients. Therefore, although it is generally accepted that a larger target dose reduction is associated with small and peripheral tumours amidst lower lung density, a clear and statistically significant trend has been challenging to demonstrate in patient studies.

The association of these patient characteristics with the magnitude of target dose reduction indeed makes intuitive sense. The dose difference comes from calculation errors by the less accurate (so-called Type-A) algorithms, such as EPL or pencil beam (PB), owing to the combination of the low-density medium and small fields causing charge particle disequilibrium near the tumour-lung interface that is not well-modeled by these algorithms. A peripheral location where the tumour is often surrounded by lung tissues, as compared with a central location in which the tumour is close to the mediastinum (soft tissues), would therefore pose a bigger modeling challenge for these algorithms, leading to a larger error. Similarly, a lower surrounding lung density would lead to larger heterogeneity and make modeling more difficult; additionally, a smaller tumour size would lead to a smaller field size and hence a larger error. However, within a population of patients, this complex network of factors co-exist together with other plan-related parameters, sometimes offsetting each other and masking the effects, which makes the identification of any trend challenging without sufficient statistical power. In addition, the classification of central vs. peripheral is anatomical, in which targets are considered peripheral if they reside greater than two centimeters from mediastinal, pulmonary, and vertebral structures; all other targets are considered central. This designation may not be a directly suitable factor for classifying dose modeling challenges. For example, the modeling error might be smaller for a peripheral tumour immediately next to the chest wall than for a central tumour within 2 cm of a bronchial tree.

Quantification of tumour location could potentially provide more clear and detailed information than the simple “central vs. peripheral” classification. Therefore, in this work we quantitatively analyzed various patient-related factors that potentially impact the magnitude of target dose errors. For the analysis, all factors were made “quantifiable”, including “tumour location”. Because grouping and sub-grouping a study cohort based on different features greatly reduces the sample size (and therefore statistical power), we used linear regression to continuously analyze each feature’s individual effect in the whole study population. Different features’ interplay was comprehensively investigated by applying multiple linear regression and artificial neural network. Using a pilot cohort of 21 lung SBRT patients, we tested the above methodologies in an attempt to build a more accurate and robust model for target dose conversion from Type-A to MC algorithms.

## Methods

### Patient simulation and treatment planning

With the approval of the University of Nebraska Medical Center Institutional Review Board, 21 patients with non-small cell lung cancer (NSCLC) treated with lung SBRT at our institution between 2011 and 2012 were randomly selected. Patient characteristics are summarized in Table 1. The patients were previously treated on a Novalis™ LINAC with an M3 multi-leaf collimator (MLC) (Brainlab AG, Feldkirchen, Germany). For the current study, the patients were replanned for a TrueBeamSTx LINAC with an HD MLC (Varian Medical Systems, Palo Alto, CA, USA), on which both PB and MC algorithms were commissioned in iPlan Version 4.5 (Brainlab AG, Feldkirchen, Germany).

**Table 1:** Patient, tumour, and treatment characteristics for the patient cohort used in our study

Parameter	Total
Patients	21 (9 central and 12 peripheral)
Median GTV, cm^3^ (range)	6.9 (0.6–6.9)
Median PTV, cm^3^ (range)	30.1 (7.2–110.2)
Median tumour (GTV) diameter, cm (range)	3.1 (1.3–5.1)

Each patient was simulated in a BlueBAG™ immobilization system (Medical Intelligence, Schwabmünchen, Germany) with a free-breathing 3D CT followed by a 4D CT on a Sensation Open CT scanner (Siemens, Erlangen, Germany) with an Anzai belt system (Anzai Medical Systems, Tokyo, Japan) as the respiratory surrogate. On iPlan, the 3D CT and 4D CT images were fused using the common frame of reference or rigidly to the spine. The gross tumour volume (GTV) was contoured as the gross disease on the 3D CT, and the internal target volume (ITV) was contoured using the 3D CT, the maximum intensity projection (MIP) from the 4D CT, and each phase of the 4D CT, as the union of the gross disease seen on these images. A 6 mm uniform expansion from the ITV then generated the PTV.

Dynamic conformal sub-arcs of 6 MV photons were employed for the treatment, combining into a 360° co-planar total arc, ignoring possible patient-machine clearance issues for large patients with very peripherally situated lesions. The 360° total arc was employed for the current study to remove gross angular dose dependence associated with a partial arc, although small angular effects might still exist due to adjustable relative weighting on the sub-arcs for plan optimisation purposes. The MLC apertures of the arcs dynamically conformed to the PTV at every 10° (but delivered continuously) with a 2 mm initial margin. The MLCs were manually adjusted and forwardly-optimised during planning to provide desirable dose coverage and conformality. RTOG relative dose-volume constraints for the target, organs-at-risk (OARs) and normal tissue were followed [2, 3].

The prescription was 48 Gy in 4 consecutive fractions, planned with the PB algorithm using iPlan 4.5. The plans were normalized so that 95 % of the PTV was covered by the prescription dose, and the prescription dose was around 90 % of the maximum dose for all cases. The plans were then re-calculated using the iPlan 4.5 MC algorithm [15]. The full MLC geometry simulation “Accuracy Optimised Model”, with a spatial resolution of 2 mm and variance of 1 %, was used.

### Dosimetric comparison and difference quantification

Both dose-volume histograms (DVHs) and isodose distributions were reviewed to compare the PB and MC calculations for each plan. For the quantitative analysis, D95 (the minimum dose received by 95 % of the volume) of the PTV and the GTV were obtained. Because the dose coverage of the targets were always overestimated by the PB algorithm as compared with the MC algorithm, the MC vs. PB percent D95 deficiency was calculated for the PTV and the GTV of each patient. According to the plan normalization, the PTV D95 by PB was the nominal prescription dose. The percent D95 deficiency values of the PTV and the GTV were selected as the surrogate outputs for the current study on factors affecting the dose conversion. The linear correlation between them was first evaluated to assess whether it was necessary to explore either both or just one.

### Patient-related feature extraction and quantification

For each patient, the following quantitative patient-related indices, potentially affecting the magnitude of target D95 deficiency, were extracted:

- Target size - PTV volume (cm^3^), GTV volume (cm^3^), MaxD (mm, defined as the maximum dimension of the GTV).
- Target location - the mean and standard deviation of the density, as surrogated by the Hounsfield Unit (HU) on the 3D CT, for the following structures: PTV, GTV, PTV margin (defined as PTV minus GTV), Shell2mm (defined as the 2-mm-thick shell structure immediately outside the PTV, which corresponded to the initial MLC aperture margin before the manual tuning of the leaf positions), Shell15mm (defined as the 15-mm-thick shell immediately outside the PTV, which corresponded to the region surrounding the PTV within the d_max_ of 6MV photons), and Shell50mm (defined as the 50-mm-thick shell immediately outside the PTV, which was randomly chosen to represent a broader region surrounding the PTV that received high to low radiation dose). If a shell structure outside the PTV went beyond the patient body contour for a peripheral lesion, only the portion inside the patient body was considered. For the density indices, the density means were used as the primary indices, and the density standard deviations were used as the secondary indices. Among all density features, the density of GTV is unlikely a true location feature - as the density of the gross tumour may be pathology rather than location dependent – but was included in this category for exploration purposes. In addition to the density indices, d_min_ (mm) was also used for target location, defined as the minimum distance from the GTV surface to the nearest soft tissue.
- Lung density - the mean and standard deviation of the lung density of the whole lungs (surrogated by the HU on the 3D CT). The lung volume did not exclude the tumour volume.

### Quantitative analysis on the feature dependence

A comprehensive, quantitative analysis on the above features and dose conversion outputs was conducted using single linear regression, multiple linear regression, and artificial neural network regression.

**Fig. 1:**
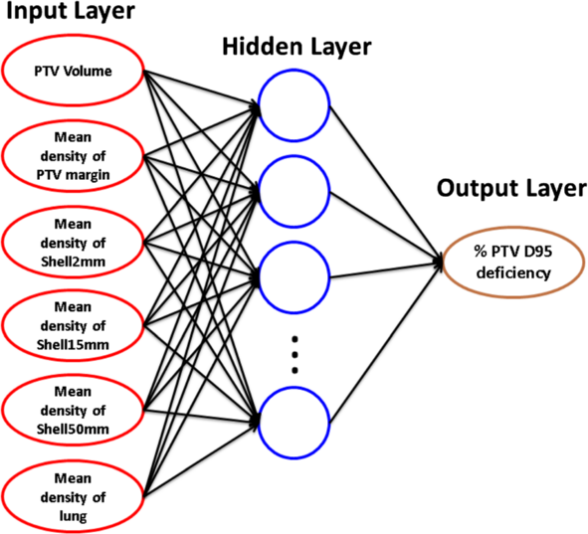
Schematic of the ANN regression setup

1. Single linear regression
  Linear regression was first used to study the correlation between the two outputs, namely between the D95 deficiency values of the PTV and the GTV. It was then used to evaluate correlations between the output and each of the above-described quantitative indices (features). The regression was conducted using Matlab, and the results were evaluated with the coefficients of determination (R^2^).
2. Multiple linear regression
  Multiple linear regression models were tested with the investigated features to identify a multiple-factor model with the highest correlation coefficient and statistically significant independent features. The regression was conducted using Matlab. The models were evaluated with the significance factor and P values for individual features.
3. Artificial neural network (ANN) regression
  This ANN regression method was implemented using the statistical language R [33]. A feed-forward neural network with a single hidden layer was constructed. This is the simplest type of ANN, in which the information moves in only one direction from the input layer through the hidden layer to the output layer. A schematic is plotted in Fig. 1 illustrating the simulation setup. In the input layer, six features were selected, which were PTV volume, mean densities of PTV margin, Shell2mm, Shell15mm, Shell50mm, and Lung. In the output layer, there was only one node, the percent PTV D95 deficiency, which was normalized into the range from 0 to 1 in the training dataset. The size of the hidden layer underwent an optimisation procedure. This neural network method was implemented using the R package, nnet, with the multinomial log-linear model. To obtain the optimal model, we employed the R package, caret, to search the parameter space and estimate the model performance for the given training dataset. The optimised parameters were the size of the hidden layer in the range of (2, 3, 4, 5, 6, and 7) and weight decay in the range of (0.001, 0.005, 0.01, 0.05, 0.1, 0.5, and 1). The root mean square of errors (RMSE) was calculated to evaluate the performance of the ANN models and compare the optimal ANN model with the multiple linear regression model.

## Results

### Dosimetric comparison between PB and MC recalculated plans

Similar to previous studies, in our patient cohort, the MC dose calculation revealed considerable target under-coverage compared with the original PB plan, yielding a median (range) of percent D95 deficiency at 15.8 % (6.1–32.0 %) for the PTV and 8.6 % (2.8–20.0 %) for the GTV, respectively. Visual inspection of the isodose distribution revealed that the target periphery was the primary region of underdosage. This was to be expected, as the uncertainty of the PB algorithm would be the largest at the periphery of the target where the tumour-lung interface caused a large density gradient. Therefore, the median (range) percent differences of the target mean and maximum dose, D_mean_ and D_max_, were smaller, at 10.4 % (4.0–24.6 %) and 4.7 % (0.9–14.5 %) for the PTV, and 6.2 % (2.9–18.6 %) and 4.7 % (0.6–14.6 %) for the GTV, respectively. The dose differences for the OARs and normal tissues were relatively small, within 3 % for all structures.

**Fig. 2:**
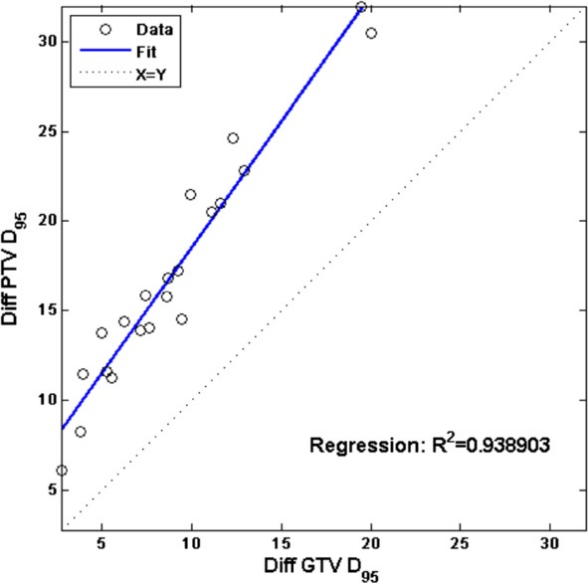
Scatter plot and linear regression between percent GTV and PTV D95 differences

Similar to the findings of previously published studies [16], the percent D95 deficiency showed a strong linear correlation between the PTV and GTV (*R*^*2*^ = 0.94), as plotted in Fig. 2. Therefore, only percent PTV D95 deficiency was used in the subsequent results to describe the target dose conversion.

### Linear regression

**Fig. 3:**
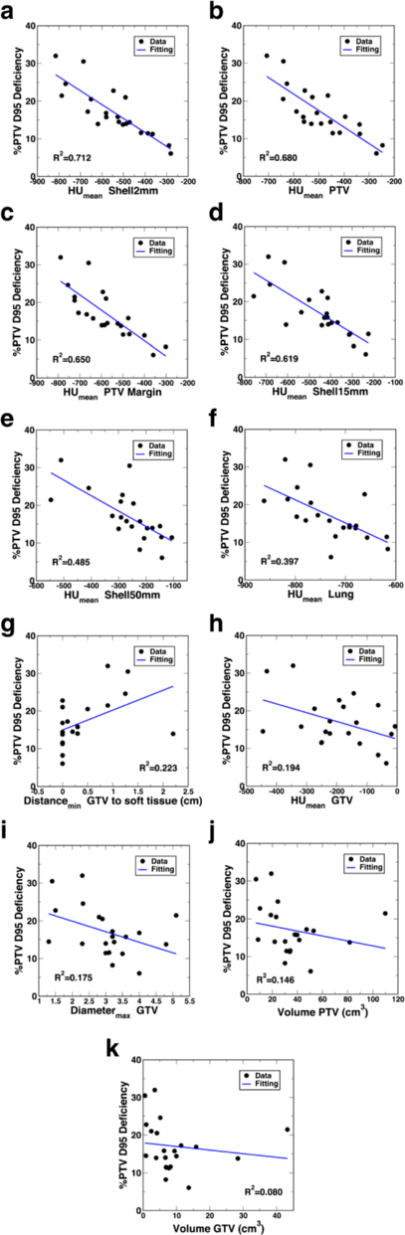
Scatter plot and single linear regression between percent PTV D95 deficiency and mean density of Shell2mm (**a**), mean density of PTV (**b**), mean density of PTV margin (**c**), mean density of Shell15mm (**d**), mean density of Shell50mm (**e**), mean density of lung (**f**), dmin (**g**), mean density of GTV (**h**), MaxD (**i**), PTV volume (**j**), and GTV volume (**k**), respectively

The linear regression results on individual quantitative features with the percent PTV D95 deficiency are plotted in Fig. 3. Among the features, the target location indices as a group showed higher correlations with the dose conversion than the target size indices and lung density. Among the target location features, all density indices, except density of GTV, demonstrated fairly high correlations with the dose conversion, while the distance index d_min_ showed a relatively weaker correlation. The lung density showed an intermediate correlation. Interestingly, the correlations between the three target size indices and the dose conversion were fairly weak.

For the percent PTV D95 deficiency, the following R^2^ values in decreasing order were found for the studied indices: mean density of shell2mm (0.71), mean density of PTV (0.68), mean density of PTV margin (0.65), mean density of shell15mm (0.62), mean density of shell50mm (0.49), mean density of lung (0.40), d_min_ (0.22), mean density of GTV (0.19), MaxD (0.17), PTV volume (0.15), GTV volume (0.08). As shown from the figures, the density of the various structures is always negatively correlated with the percent PTV D95 deficiency. This is to be expected, because the lower the density outside the tumour - thus relating to higher density heterogeneity between the tumour/surrounding - the more difficult it is for the PB algorithm to calculate target dose, thus leading to larger uncertainty or dose deficiency. Out of these structures, the density of the shell2mm structure, whose diameter corresponds to the initial block/MLC margin used for planning before manual adjustments, showed the strongest correlation. It can also be noted on this plot that the linear regression fitting worked better in the higher density region than the lower density region. Densities of PTV, GTV-to-PTV margin, and the shell15mm structure also showed strong correlations, which would indicate that the non-soft-tissue components of the PTV, as well as the region within the photon dmax surrounding the PTV, had more influence on the PB algorithm target dose calculation uncertainty than the inner components of the PTV (such as GTV) and the farther region surrounding the PTV (such as the shell50mm structure and lung). For the other non-density indices, as expected, the PTV and GTV sizes as well as the maximum GTV diameter all showed negative correlations, while the minimum distance between GTV and the nearest soft-tissue showed a positive correlation with the percent PTV D95 deficiency. However, the correlations were all relatively weaker as compared with the density-location indices.

On the linear regression analysis of the secondary density indices, the density standard deviations of the various studied structures showed varying degrees of correlation. Regression with percent PTV D95 deficiency calculated R^2^ values for density standard deviations as listed in descending order: Shell50mm (0.50), Shell2mm (0.48), GTV (0.39), PTV margin (0.36), PTV (0.15), lung (0.06),and Shell15mm (0.01).

### Multiple linear regression

Different feature combinations were tested in multiple linear regression models. The most accurate model for dose conversion was established with two independent indices: the mean density of shell2mm and the PTV volume. Equation 1 describes the model:

1 $$ \%Dif\ {f}_{PTVD95}=-0.6623 - 0.08136\ *\ {V}_{PTV} - 0.03784\ *\ {\overline{HU}}_{Shell2 mm} $$

in which % *Dif f*_*PTVD*95_ is the predicted percent PTV D95 deficiency, *V*_*PTV*_ is the volume of the PTV in cubic centimeters, and ${\overline{HU}}_{Shell2 mm}$ is the mean Hounsfield Units of the 2-mm-thick shell structure immediately outside the PTV. For this model, a high significance factor of 5.3 × 10^−7^ was calculated, with statistically significant P values for the two parameters (*P* = 0.012 for the PTV volume and *P* = 1.9 × 10^−7^ for the mean density of shell2mm).

Adding other variables to this model, such as the mean densities of PTV, GTV, lung, or PTV margin, decreased the significance factor of the model, and decreased the statistical significance of the original two variables. The added variables also did not show statistical significance (*P* > 0.05).

A slight improvement of the model was achieved by replacing the PTV volume feature with its surface area feature. The rationale was that the surface area, more than the volume, affected the heterogeneous interface and dose modeling error. The refined model is described in Eq. 2.

2 $$ \%Dif\ {f}_{PTVD95}=1.62022 - 0.45734\ *\ {V_{PTV}}^{2/3} - 0.03705\ *\ {\overline{HU}}_{Shell2 mm} $$

The refined model yielded a higher significance factor of 3.0 × 10^−7^, with *P* values of 0.006 and 1.6 × 10^−7^ for the two model parameters.

### Artificial neural network (ANN) regression

To explore the parameter space and identify the global optimum, the ANN regression algorithm was employed to test the combinations of the six selected features (six predictors) for this machine-learning platform. Using RMSE to select the optimal model for the ANN regression, the optimal model was identified with the size of ANN nodes of 4 and the decay of 1.005. Using leave-one-out cross validation on the 21 samples, the average normalized RMSE was 0.072 with a standard deviation of 0.029, which did not significantly improve upon the multiple linear regression model at an average normalized RMSE of 0.091 with a standard deviation of 0.018 (*P* = 0.381 in a Mann–Whitney *U* test between the square errors calculated by the two models). The results of ANN regression further proved that the simple multiple linear regression model based on the two factors described in Eq. 2 was robust and near optimal for all potential combinations from the six parameters.

## Discussion

Previous reports have identified target size, target location, and lung density as the three most prominent patient factors that affect the target dose conversion, i.e. the target dose calculation error by Type-A algorithms [16, 17, 30], although sometimes a statistical difference was not found between groups [28]. The underlying challenge is the complexity of the involved factors and their interplay; a large sample size is necessary to establish sufficient statistical power while the sample size is reduced in folds through grouping and subgrouping based on features in these kinds of investigations.

**Fig. 4:**
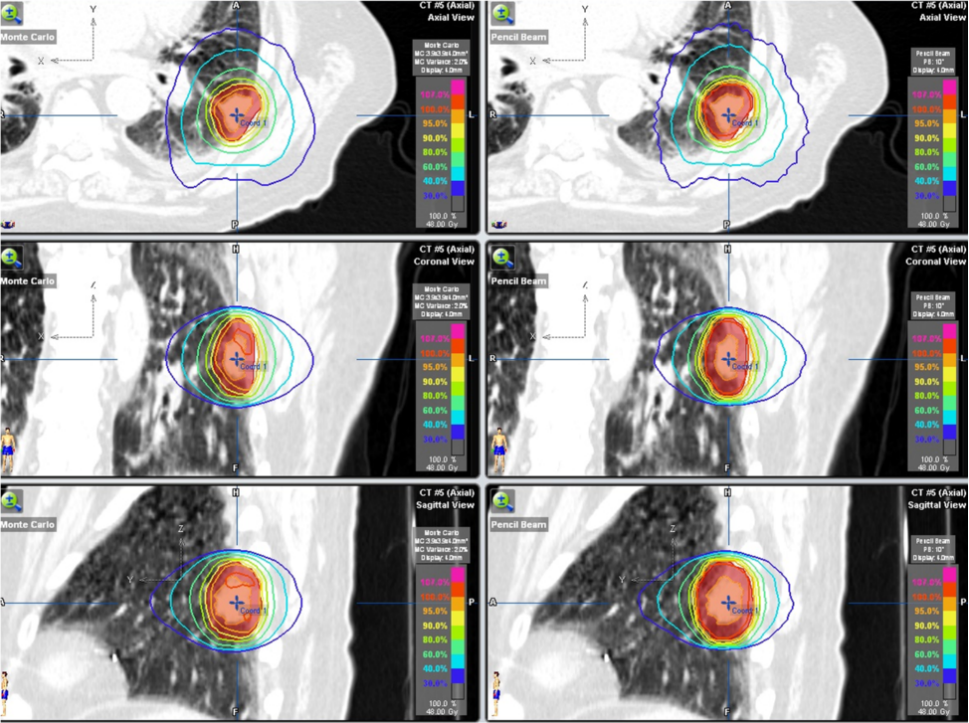
Isodose distribution comparison between MC (*left*) and PB (*right*) dose calculations for one example patient. The calculated percent PTV D95 difference was only 8.7 %, the second lowest among our 21 patients, for this peripheral tumour due to the close proximity of the tumour to the chest wall

To exacerbate the problem, some grouping mechanisms may introduce ambiguity. For example, the location grouping of central and peripheral tumours was historically introduced based on the consideration of treatment toxicity. In the context of dose calculation error in the heterogeneous media, this grouping method does not always capture intrinsic differences. A peripheral tumour is more likely to be an “island” tumour, or a tumour completely surrounded by low density lung tissue, than a central tumour would, leading to higher target dose calculation errors. However, a peripheral tumour may also be close to high density tissues, such as the chest wall, therefore leading to similar or even less target dose errors than a central tumour. Figure 4 shows one such example, comparing PB- and MC-calculated dose distributions. Because the tumour is attached to the chest wall, the target dose difference is mostly on the lung side and consists of a small magnitude. The percent PTV D95 difference between the PB and MC calculations for this patient was only 8.7 %, the second lowest among our 21 patients. Therefore, we chose to use the mean densities of the various target-related structures as quantifiable and unambiguous location features.

To our knowledge, this is the first instance of such a method being proposed in this context. Using all quantifiable features related to tumour location, tumour size and lung density, we could systematically assess the target dose difference dependence on these features, as well as study the interplay among these features. Our study found that tumour location, as surrogated by our proposed mean density features, was the most influential factor on the target dose difference. Tumour size, on the other hand, showed much lower linear correlation. For target location, the density features were also much more sensitive than the distance feature, dmin, possibly due to the fact that the dmin values among the patients were not well-differentiated. In addition, the distance feature samples in only one direction, the direction with the nearest soft-tissue to the tumour edge. In contrast, the density features sample in all directions, and therefore better represent the location information related to target dose calculation uncertainty.

By applying a Mann–Whitney *U* test on the PTV D95 differences to compare between the central and peripheral groups among our 21 patients, with the hypothesis that the two groups were significantly different, we calculated a *P* value of 0.352 which rejected the hypothesis. In contrast, our density-location features calculated highly significant correlations in single linear regression. For example, the mean density of Shell2mm yielded a correlation coefficient of −0.84, a much stronger correlation than the threshold of −0.55 at *P* = 0.01 for our sample size. These results indicated that the mean density designation of location better extracted the location feature than the conventional dichotomy of central and peripheral in the context of target dose conversion between algorithms for lung SBRT.

**Fig. 5:**
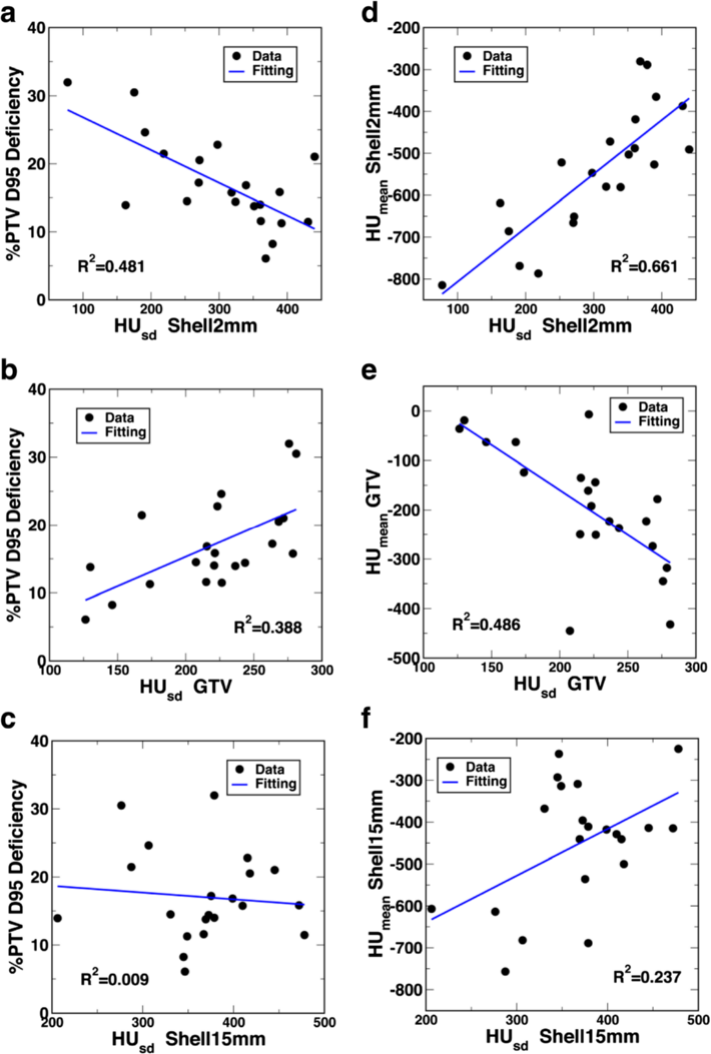
The left panels show the scatter plot and single linear regression between percent PTV D95 difference and density standard deviation of Shell2mm (**a**), GTV (**b**), and Shell15mm (**c**); and the right panels show the corresponding scatter plot and single linear regression between the density standard deviation and the density mean of Shell2mm (**d**), GTV (**e**), and Shell15mm (**f**)

In addition to using the mean density features for location, we also examined how the variability of density affected the dose error through the investigation on density standard deviation features. Interestingly, the results were largely different among different structures. Figure 5a–c show the scatter plots for 3 example structures: Shell2mm, Shell15mm, and GTV. It was first surprising to note that, while the density standard deviation of Shell2mm showed a negative correlation with the dose conversion, that of GTV showed a weaker (yet positive) correlation, and that of Shell15mm showed almost no correlation at all. These somewhat unexpected observations became apparent after the correlations between the density standard deviations and their corresponding density means were plotted in Fig. 5d–f. For Shell2mm, the density standard deviation positively correlated with the density mean. This region immediately surrounds the PTV. A lower mean density in Shell2mm was more likely a PTV surrounded only by lung, and hence had a smaller standard deviation. A higher mean density, on the other hand, was more likely a PTV surrounded by both lung and soft tissue, and hence had a larger standard deviation. In contrast, for GTV, the correlation was the opposite. A higher mean GTV density was more likely to be a more homogeneous, solid GTV, and a lower mean GTV density more likely contained greater low-density tissue components and hence greater tissue heterogeneity (larger density standard deviation). The lack of correlation between the density mean and standard deviation in Shell15mm could explain the lack of a correlation between the density standard deviation and the dose conversion.

**Fig. 6:**
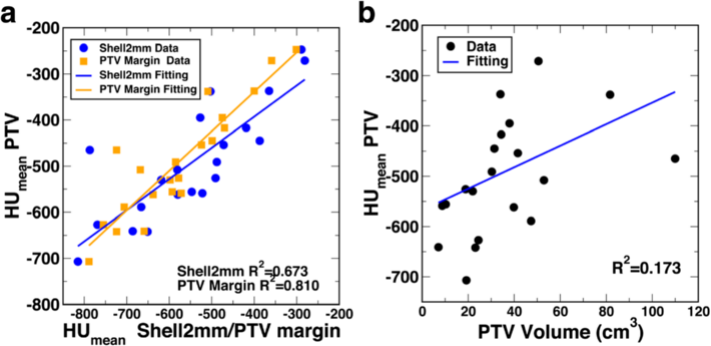
**a** Scatter plot and single linear regression shows fairly strong correlation between the mean density of PTV and that of PTV margin and Shell2mm. **b** Scatter plot and single linear regression shows the lack of correlation between the mean density of PTV and the volume of PTV

The quantifiable features designed for our study allowed the examination on these features’ interplay through multiple factor regression analysis using both multiple linear regression and ANN regression. The optimal model generated from multiple linear regression had two features, one density-location feature, ${\overline{HU}}_{Shell2 mm}$, and one feature derived from target size, *V*_*PTV*_^2/3^. It was initially somewhat unexpected to find that adding additional seemingly independent features that had high single linear regression R^2^ values, such as mean densities of PTV or PTV margin, decreased the performance of the model. This observation was examined by plotting the mean densities of the various structures against the PTV density. As shown in Fig. 6a, there was actually a relatively strong positive linear correlation between these density features. In contrast, a volume feature such as the PTV volume, although by itself showed low correlation on linear regression, strengthened the multiple regression model because of its independence as shown in Fig. 6b. One possible limitation of our model was that Shell2mm, which showed the strongest single linear correlation and was hence used as the location feature in the multiple model, corresponded to the initial MLC block margin size used in our plans. While this may be specific to our plans, the strong correlations shown in Fig. 6a between the density-location features demonstrated that any other more generic location feature such as the density of PTV or PTV margin, could therefore replace Shell2mm and still work relatively well in the multiple-factor model.

In addition to revealing the quantitative dependence relationship between the magnitude of the target dose difference and the comprehensive list of features investigated in our study through single-factor analyses, our multiple-factor model and proposed methodology could potentially facilitate the challenging yet necessary process of understanding the relationship between historical outcomes and true target dose to establish a proper prescription guideline for the new MC-based paradigm. The best approach for such an effort may be to systematically review historical data and analyze, on a case-by-case basis, the correlation between the “true” target dose received based on the accurate MC recalculation of the original treatment plan and the clinical outcomes. This way it can be ascertained whether target underdoses due to calculation errors related to clinical failure. With this knowledge, the “minimum curable target dose” may be established using the accurate MC calculation. However, this type of studies are difficult to conduct because most historical cases with clinical outcomes may have been planned using old treatment planning systems that have since been retired from clinical operations, do not have MC algorithms available, or may have been treated on retired LINACs that prevent the commissioning of newly available MC algorithms. Additionally, because of the low local recurrence rates associated with lung SBRT, studies with very large sample sizes are likely required to yield meaningful information. For example, in a study with a large cohort of 82 tumours [18], though able to show a significant correlation between target dose and local control, the target dose differences came from the original prescription differences rather than the Type-A algorithm target dose calculation errors involved in the original prescriptions. On these regards, our model or other models generated with similar methodologies as ours could be easily applied to a very large number of historical cases and scale up the meaningful investigations on clinical outcomes vs. true target dose.

In our study, the advanced machine learning method, ANN regression, did not significantly improve upon the simple 2- factor multiple linear regression model. Aside from the satisfactory performance of the multiple linear regression model, the accuracy of the ANN regression might also be limited by the small size of our training data set (21 patients), largely because a machine-learning algorithm requires a large volume of training data.

Another limitation of our study is the possible dependence of our quantitative results on the specifics of our plans. For example, the Shell2mm structure corresponds to the 2 mm initial MLC block margins used in our plans, although as discussed above, the other non-specific structures such as PTV or PTV margin could easily replace Shell2mm and still create accurate models. The quantitative results may also be influenced by other specifics of our plans, such as beam energy, planning and delivery technique, and prescription isodose line. Firstly, our plans used 6 MV beams, which would result in lower target dose differences based on different algorithms than would higher energy beams such as 10 MV, and higher differences than would lower energy beams such as 6 MV in flattening-filter-free mode. Secondly, our plans used dynamic conformal arcs, which may exhibit similar quantitative dependence as plans with multiple conformal beams, but may be quite different from intensity-modulated treatments such as intensity-modulated radiation therapy (IMRT) and volumetric-modulated arc therapy (VMAT), wherein the target coverage is created by an ensemble of small beamlets. However, because we are mostly interested in better modeling the dose difference from the earlier era from which most historical data were generated, dynamic conformal arcs and multiple conformal beams may indeed be the most relevant treatment techniques. A similar limitation of our study is the margin-based motion management used for our plans, i.e. the ITV-based target definition. Because the amount of low-density margin impacts the magnitude of the algorithm-related target dose difference, as shown by our study, the target differences in our study would be higher than other motion management methods requiring less target margin such as tracking. This may render our quantitative model not directly applicable to some CyberKnife datasets [9, 12, 17, 26, 28]. Lastly, while the plans in our study had fairly homogeneous target dose (prescribed at about 90 % of the maximum dose) based on our own clinical practice, larger target dose heterogeneity is allowed and has been used for lung SBRT. For example, RTOG protocols accept a wide range of prescription isodose lines between 60 % and 90 % [2, 3]. A recent study demonstrated the dependence of algorithm-related target dose differences on this planning parameter [34], indicating a higher sensitivity to dose algorithms for plans using a more homogeneous target dose prescription. Therefore, the absolute magnitudes of target dose differences in our study would be lower if more heterogeneous target dose prescriptions were used instead. On the other hand, while the quantitative model from our study may not directly apply to clinical cases with the above-mentioned planning/delivery specifics, our proposed methodology is easily transferable and applicable to those data sets.

**Fig. 7:**
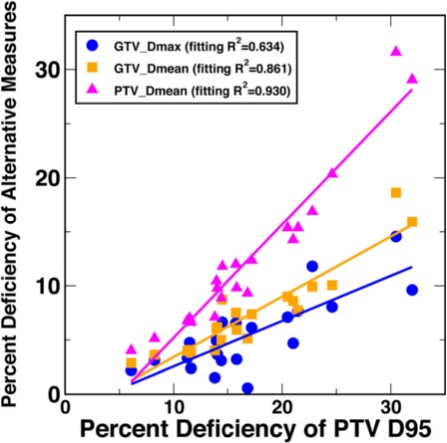
Scatter plot and single linear regression shows the correlations between the percent PTV D95 deficiency and the percent deficiency of alternative prescription measures-PTV mean dose, GTV mean dose, and GTV max dose, respectively

In our study, we explored D95 of PTV and GTV as the model outputs. After identifying a high linear correlation between PTV D95 and GTV D95, we reported in this paper the results based on PTV D95 as the model output. While this was a reasonable choice, as PTV D95 has been (and still is) the standard prescription practice for lung SBRT, a few recent studies have proposed alternative prescription concepts such as GTV maximum and GTV mean doses [9, 12, 16, 35, 36]. As shown herein, with centralized maximum dose planning concepts, such parameters – especially the maximum dose – are less sensitive to algorithm-related target dose differences than peripheral dose parameters such as D95. Also, GTV may be a dosimetrically more suitable surrogate than PTV especially with algorithms such as MC that can accurately handle heterogeneity in dose calculations, because the low-density tissue in the PTV margin does not accurately predict dose to the dense tumor. While these concepts still have some unresolved practical challenges, such as a way to ensure the geometric coverage provided by the conventional PTV concept in plan optimization, recent studies have already started to establish the dose–response relationship based on such concepts. For example, Guckenberger et al. [35] reported a retrospective multi-institutional study on 399 patients with stage I non-small cell lung cancer and 397 patients with 525 lung metastases, in which the local control was shown to explicitly depend not only on PTV prescription dose, but also significantly on GTV maximum and mean doses. To preliminarily explore these alternative prescription concepts, we also analyzed the correlations of PTV D95 to the mean and maximum doses of PTV and GTV, and conducted quantitative analyses of the studied patient factors using these latter target dose surrogates as the output endpoints in our datasets. Unsurprisingly, the maximum doses of PTV and GTV were virtually the same in plans with centralized maximum dose concepts. As plotted in Fig. 7, linear correlation with R^2^ values of 0.93, 0.86, and 0.70 were calculated between PTV D95 and PTV mean dose, GTV mean dose, and GTV maximum dose, respectively. As expected, these alternative endpoints showed weaker dependence on the target location and volume features than PTV D95, with the weakest for GTV maximum dose. Correlation with target location features was still statistically significant in multivariate analyses for these endpoints, with the strongest linear correlation R^2^ values with PTV density at 0.65, 0.64 and 0.60 for PTV mean dose, GTV mean dose, and GTV maximum dose, respectively.

In summary, because of the different levels of dose calculation uncertainty for PB at different target locations (hence involving different levels of heterogeneity), the quantitative dependence for these alternative prescription concepts may be different from PTV D95 studied in our work. It is possible that these new prescription concepts may lead to a complete paradigm shift for future lung SBRT applications. However, currently the PTV D95 based prescription still represents the mainstay of standard clinical practices. Furthermore, an accurate target dose understanding of the current and historical plans using this prescription concept, such as those provided by our current model and methodology, will also serve as the foundation for future research to compare with the newer, alternative prescription concepts.

## Conclusion

Using 21 lung SBRT patients, our pilot study for the first time quantitatively analyzed the dependence of the target dose differences between PB and MC dose calculations on various patient factors such as target size, location, and lung density. We also applied multiple linear regression and artificial neural network to establish continuous models to predict the dose conversion ratio based on these factors. The target location indices were found to have the largest influence on the dose conversion, with *R*^*2*^ > 0.6 in linear regression for the mean densities of the PTV, the GTV-to-PTV margin, and the high-dose region immediately outside the PTV. The multiple-factor models yielded high significance factors. Further studies are warranted to refine the models based on larger patient populations, for which machine learning methods may provide additional improvements, and apply the improved models to retrospectively analyze large-scale historical data to potentially correlate accuracy of PB dose prescriptions and clinical outcomes.
